# Regulation of Gγ-Globin Gene by ATF2 and Its Associated Proteins through the cAMP-Response Element

**DOI:** 10.1371/journal.pone.0078253

**Published:** 2013-11-06

**Authors:** Li Liu, Subhradip Karmakar, Ruby Dhar, Milind Mahajan, Alina Choudhury, Sherman Weissman, Betty S. Pace

**Affiliations:** 1 Department of Molecular and Cell Biology, The University of Texas at Dallas, Richardson, Texas, United States of America; 2 Institute for Genomics and Systems Biology, University of Chicago, Chicago, Illinois, United States of America; 3 Department of Genetics and Genomic Sciences, Icahn School of Medicine at Mount Sinai, New York, New York, United States of America; 4 Department of Genetics, Yale University, New Haven, Connecticut, United States of America; 5 Department of Pediatrics, Georgia Regents University, Augusta, Georgia, United States of America; Southern Illinois University School of Medicine, United States of America

## Abstract

The upstream Gγ-globin cAMP-response element (G-CRE) plays an important role in regulating Gγ-globin expression through binding of ATF2 and its DNA-binding partners defined in this study. ATF2 knockdown resulted in a significant reduction of γ-globin expression accompanied by decreased ATF2 binding to the G-CRE. By contrast, stable ATF2 expression in K562 cells increased γ-globin transcription which was reduced by ATF2 knockdown. Moreover, a similar effect of ATF2 on γ-globin expression was observed in primary erythroid progenitors. To understand the role of ATF2 in γ-globin expression, chromatographically purified G-CRE/ATF2-interacting proteins were subjected to mass spectrometry analysis; major binding partners included CREB1, cJun, Brg1, and histone deacetylases among others. Immunoprecipitation assays demonstrated interaction of these proteins with ATF2 and *in vivo* GCRE binding in CD34^+^ cells undergoing erythroid differentiation which was correlated with γ-globin expression during development. These results suggest synergism between developmental stage-specific recruitments of the ATF2 protein complex and expression of γ-globin during erythropoiesis. Microarray studies in K562 cells support ATF2 plays diverse roles in hematopoiesis and chromatin remodeling.

## Introduction

Fetal γ-globin gene silencing accompanied by adult β-globin activation results in the switch from fetal hemoglobin (HbF) to adult hemoglobin synthesis after birth [1]. Mechanisms underlying globin gene expression have been studied extensively, demonstrating that both *trans*-activators and repressors are involved in γ-globin regulation [2]–[9]. Furthermore, mutations in the β-globin gene result in different hemoglobinopathies such as sickle cell anemia and β-thalassemia. An effective therapeutic strategy for these disorders is to increase HbF levels to ameliorate clinical symptoms [10].

Previous studies from our laboratory focused on the role of a cAMP response element (TGACGTCA) at −1222 in the Gγ-globin promoter (G-CRE). The G-CRE is a *cis*-acting element regulating Gγ-globin in response to p38 MAPK signaling [11], [12]. Moreover, transcription factors such as CREB1, cJun and activating transcription factor 2 (ATF2) bind the G-CRE to enhance Gγ-globin expression [11]–[13]. These factors are members of the basic leucine zipper (bZIP) family of transcription factors. Once activated ATF2 forms a homodimer or heterodimers with cJun, or other AP1 family members and binds CREs to regulate diverse target genes including CREB1, ATF, and Jun/Fos family members [14], [15].

The goal of this study was to determine the physiologic role of ATF2 in γ-globin regulation and to characterize the multi-protein complex that binds the G-CRE. Employing affinity chromatographic purification, we identified ATF2 DNA-binding protein partners. Chromatin immunoprecipitation (ChIP) assay demonstrated components of the ATF2-protein complex are recruited to the G-CRE in primary erythroid progenitors. Subsequent microarray analysis of ATF2-specific siRNA (siATF2) treated K562 cells identified potential downstream targets involved in γ-globin regulation and hematopoiesis.

## Materials and Methods

### Antibodies

Antibodies for ATF2 (sc-242), CREB1 (sc-240), cJun (sc-74543), p-cJun (sc-16312), hnRNP C1/C2 (sc-32308), and TFIID (TBP, sc-204) were purchased from Santa Cruz Biotechnology (Santa Cruz, CA). Antibodies for Brg1 (07–748), HDAC2 (05–814), acetyl Histone H3 (07–352) and Actin (MAB1501) were purchased from Millipore (Billerica, MA). Horseradish peroxidase (HRP)-conjugated goat anti-mouse (31432) and goat anti-rabbit (31462) antibodies were purchased from Thermo Fisher Scientific Inc. (Rockford, IL). Antibody against the FLAG epitope (M2) was obtained from Sigma-Aldrich (St. Louis, MO).

### Cell Culture and Treatments

K562 cells were purchased from ATCC (Manassas, VA) and maintained in Iscove’s Modified Dulbecco’s medium (IMDM) containing 10% fetal bovine serum (FBS, Atlanta Biologicals, Atlanta, GA), supplemented with penicillin (100 U/ml) and streptomycin (0.1 mg/ml). For treatment with chemical compounds, cells were incubated with 2 mM NaB for 48 hours.

For primary erythroid progenitor cultures, human peripheral blood mononuclear cells were purchased from Carter BloodCare (Fort Worth, TX) in accordance with guidelines of the Institutional Review Board at the University of Texas at Dallas. Erythroid progenitors were generated using the two-phase liquid culture system as previously published [16]. CD34^+^ stem cell were purchased from the Yale Center of Excellence in Molecular Hematology core facility; cells were collected from the peripheral blood of healthy donors after G-CSF stimulation in accordance with guidelines of the Institutional Review Board at Yale University. The CD34^+^ cells were cultured following a previously published protocol [17].

### Plasmid Constructs and Stable Cell Lines

Total RNA isolated from K562 cells was reverse-transcribed using Superscript VILO from Invitrogen (Grand Island, NY). The full length ATF2 coding sequence flanked by *NheI* and *SalI* sites at the 5′ and 3′ end respectively was cloned into the pCI-Neo 6X(His) vector. Recombinant constructs were confirmed by direct sequencing and transfected into K562 cells to select for stable cell lines in the presence of G418. Expression of His-FLAG-tagged ATF2 was confirmed by western blot.

### Transient Transfections

Transfection of wild type K562 cells or ATF2 stable lines with siATF2 (M-009871-00-0005) along with non-targeting negative control Scrambled siRNA (D-001210-01-05) was conducted using the DharmaFECT 1 transfection reagent (T-2001) purchased from Thermo Scientific Inc. per the manufacturer’s instructions. Transfected cells were incubated for 72 hours in triplicate with 20 nM or 50 nM siATF2 and 50 nM of scrambled siRNA. Transfections of siRNA in primary erythroid progenitors were conducted at day 11 using the CD34^+^ Nucleofector kit (DPA-1003) per the manufacturer’s instructions (Lonza, Walkersville, MD) on the Amaxa Nucleofector device using program U-008. pMaxGFP plasmid (1 µg) was included to monitor transfection efficiency. After nucleofection, erythroid progenitors were cultured in phase 2 medium for 72 hours after which RNA and protein were isolated for analysis.

### Preparation of Cellular Extracts

Nuclear proteins were prepared as previously published [18]. To prepare whole cell lysates, transfected cells were lysed in buffer containing 25 mM HEPES, pH 7.9, 300 mM NaCl, 1.5 mM MgCl_2_, 0.2 mM EDTA, 0.5% Triton X-100, 3 mM DTT, 30 mM β-glycerophosphate, 1 mM sodium orthovanadate, and a protease inhibitor cocktail (Roche, Indianapolis, IN).

### Western Blot Analysis

For western blot analysis, 50 µg of protein were resolved on SDS-polyacrylamide electrophoresis (SDS-PAGE) gels, transferred to nitrocellulose, and incubated with primary antibodies. Membranes were incubated with HRP-conjugated secondary antibody (Thermo Scientific Inc.), the film developed with an enhanced chemiluminescence reagent (GE Biosciences) and images captured on X-ray film; band intensities were quantified using ImageJ software. Subsequently, the membrane was stripped by standard methods and probed with control antibodies.

### Reverse Transcription-Quantitative Polymerase Chain Reaction (RT-qPCR)

Total RNA was extracted from cells using RNA Stat-60 (TEL-TEST “B” Inc., Friendswood, TX) as previously published [19], [20]. Real-time qPCR reaction was conducted with 10 pmol of gene-specific primers (Table S1) and the glyceraldehyde-3-phosphate dehydrogenase (GAPD) gene was used as an internal control; hypoxanthine phosphoribosyltransferase 1 (HPRT1) gene expression was used as an internal control for microarray studies.

### Electrophoretic Mobility Shift Assay (EMSA)

Protein samples were incubated with 0.1 pmol of ^32^P-end labeled double-stranded G-CRE probe (5′ CCAGAGTTTCTGACGTCATAATCTACCAAGG 3′) in the reaction buffer containing 1 µg of polydIdC, and 12% glycerol. Samples were resolved on a 5% native PAGE followed by autoradiography. To identify the ATF2-containing complex, a supershift experiment was conducted with IgG or ATF2 antibody and protein prior to addition of the G-CRE probe.

### Determination of HbF Levels by Enxyme-linked Immune Assay (ELISA)

Total protein was extracted from cells after treatment and cellular HbF and total hemoglobin (Hb) were quantified using the human HbF and total human hemoglobin ELISA Quantitation kits (E80–136 and E80–135, Bethyl Laboratories, Montgomery, TX) as previously published [21]. Hemoglobin concentrations were determined using the BioTek microplate reader at 450 nm. The relative HbF levels were calculated by normalizing HbF level by the total hemoglobin and protein.

### Purification of a G-CRE-associated Protein Complex

To define the protein complex associated with the G-CRE, biochemical purification approach was performed using nuclear extracts prepared from K562 cells stably expressing His-FLAG-tagged ATF2. During the purification process, the elution fractions at each chromatographic step were tested for the G-CRE DNA binding activity and the presence of ATF2 in the fractions was monitored by western blot. Nuclear proteins were applied to a heparin sepharose column and 0.6 M NaCl elution fractions were applied to a HisTALON column (Clontech, Mountain View, CA). Eluted fractions with the presence of imidazole were applied to a column containing anti-FLAG antibody agarose resin. Fractions eluted from the FLAG column in the presence of the FLAG peptide (Santa Cruz Biotechnology) were loaded on a G-CRE oligonucleotide-affinity column after pre-clearing with a mutant G-CRE agarose matrix. The G-CRE-bound proteins were eluted using 0.4 M NaCl buffer. Proteins fractions (40–80 µg) were loaded on 10% SDS-PAGE gels and stained with colloidal blue. The bands were excised and processed for mass spectrometry analysis as previously described [22].

### Sucrose Gradient Protein Fractionation

A 10–30% w/v sucrose gradient was prepared in a 5 ml bed volume following the previously described protocol [23], [24] with some modifications [22]. Sucrose solutions (10% and 30%) were prepared in the HEMG buffer containing 25 mM HEPES-KOH, pH8.0, 0.1 mM EDTA, 12.5 mM MgCl_2_, 10% glycerol, 0.1% NP-40, 0.5 mM DTT, and 5 mM PMSF; unless indicated, all operations were performed at 4°C. The sucrose gradient was prepared with 30% sucrose, followed by 27.5%, 25%, 20%, 15% and 10% from the bottom to top of the tube. The 0.6 M NaCl eluted fractions from heparin sepharose were dialysed against the HEMG buffer. Protein fractions and molecular weight markers were loaded separately on top of the gradient and centrifuged. Fractions were collected at 0.5 ml/fraction and stored in a −80°C freezer.

### Immunoprecipitation (IP)

Nuclear extracts (500 µg) isolated from K562 cells were incubated with 2 µg of antibodies in IP buffer followed by addition of 30 µl protein A/G-agarose beads overnight. The IPs were resolved on a SDS-PAGE gel and analyzed by western blot [18]. For CD34^+^-derived erythroid cells, co-IP was performed from cells undergoing erythropoietin-induced differentiation at Day 3, 7, 14 and 21. Cells were washed twice with cold phosphate buffered saline containing protease and a phosphatase inhibitor cocktail (Roche). Cells were then lysed in buffer (Active Motif, Carlsbad, CA) and briefly sonicated; 50 µg of protein each was used to perform co-IP.

### Chromatin Immunoprecipitation (ChIP) Assay

For K562 cells, ChIP assays were conducted as previously published [11]. Immunoprecipitation was carried out with 300 µl of chromatin and 3 µg of control IgG, or specific antibodies overnight. Purified chromatin was quantified by qPCR to measure G-CRE and DNase I hypersensitivity site 2 (HS2) chromatin enrichment using primers listed in Table S1.

For the CD34^+^ cell culture, ChIP assays were performed following the protocol as previously described [25]. Purified CD34^+^ cells were differentiated with erythropoietin. At day 3, 7, 14 and 21 of erythropoietin treatment, cells were cross-linked in 1% formaldehyde followed by sonication in 1X RIPA buffer containing 0.5% SDS. Chromatin was sheared to size spanning 300–800 bp. Pre-cleared chromatin was mixed with specific antibody (10 µg) and incubated overnight with gentle rocking. The following day, the sample was incubated with protein G dynal beads (Invitrogen) and processed following standard protocols.

qPCR was performed using 1 µL of the ChIP-enriched DNA using SYBRGreen master mix (Applied biosciences). Primers for the G-CRE region were used to probe for target chromatin enrichment normalized with IgG as a random nonspecific background DNA. ΔΔCT method was used to calculate the fold change and results are represented as the mean of two biological replicates with each having two technical replicates.

### Oligonucleotides Pull-down Assay

Pull down assays were conducted as previously published [19] to determine protein-DNA interaction *in vitro*. Briefly, the 5′ biotinylated wild-type G-CRE (G-CREwt: 5′ biotin-CCAGAGTTTCTGACGTCATAATCTACCAAGG 3′) and mutant G-CRE (G-CREmt: 5′ biotin-CCAGAGTTTCTGTGGTCATAATCTACCAAGG 3′) oligonucleotides were synthesized by Sigma-Aldrich. Boxed nucleotides indicate the dinucleotide mutations. For the pull-down assays, 500 µg of nuclear extract was incubated with 1.25 µg of DNA probes in the pull-down buffer for 90 minutes. Streptavidin agarose beads (20 µl) were added overnight and precipitates were detected by western blot.

### Microarray Analysis and qPCR Confirmation

Microarray analysis was performed following the Yale Center for Genomic Analysis protocol using RNA isolated from the negative control scramble and siATF2 treated cells. Single stranded cDNA was synthesized by reverse transcription and was converted into double-stranded cDNA with subsequent purification using the Illumina TotalPrep RNA Amplification Kit. Hybridization of biotinylated cRNA to the array was conducted at 58°C followed by chip staining with streptavidin-Cy3. The chips were scanned using the Illumina BeadArray reader and analyzed using the Beadstudio software. Linear Models for Microarray Data [26] software was used to identify differentially expressed genes p<0.05 after Benjamini-Hochberg adjustment. Genes that were differentially expressed between siATF2 treated K562 cells and scrambled siRNA were divided into down-regulated and up-regulated categories. Genes that showed a minimum of 1.2 fold change or higher with respect to the control were further analyzed for pathway enrichment using IPA (Ingenuity Pathway Analysis, Ingenuity System). Verification of a subset of up- and down-regulated genes was performed by RT-qPCR with specific primers listed in the Table S1. These data are available through the National Center for Biotechnology Information Gene Expression Omnibus using accession number GSE50165.

### Statistical Analysis

Each experiment was repeated three to five times independently. Data are shown as the mean ± standard error of the mean (SEM). The unpaired student’s *t*-test was performed to determine the statistical significance; p<0.05 was considered significant.

## Results

### ATF2 Regulates γ-globin Expression in K562 Cells

**Figure 1:**
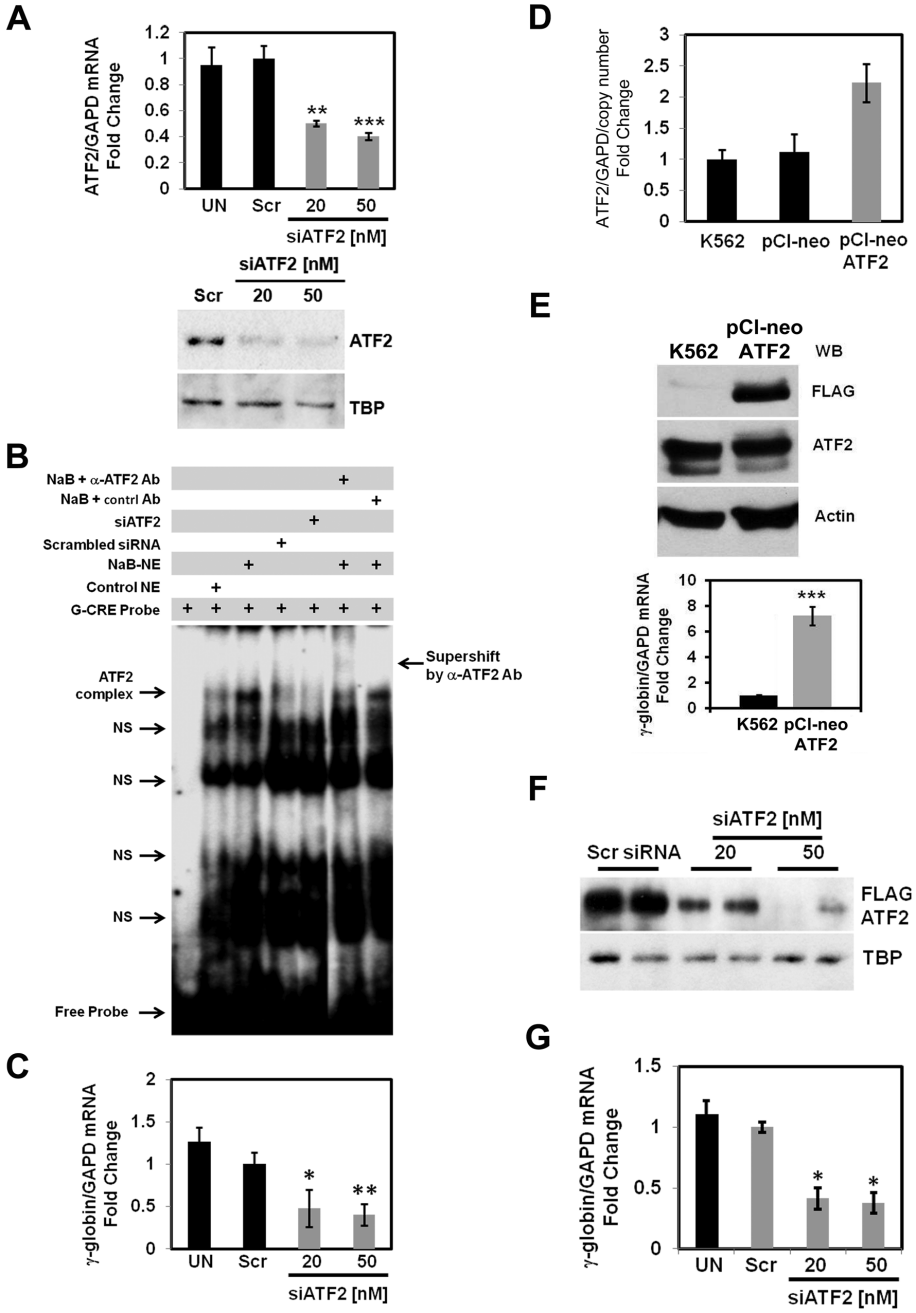
siATF2 treatment decreases γ-globin gene expression in K562 cells. (**A**) Transient transfections of K562 cells with a scrambled siRNA molecule (Scr, 50 nM) or siATF2 were performed. Total RNA and whole cell lysates were prepared and subjected to RT-qPCR and western blot respectively. The top graph shows the ATF2 levels for the mean ± SEM from three independent experiments. **p<0.01 and ***p<0.001. Shown on the bottom is a representative gel for western blot with the antibodies indicated. (**B**) EMSA assay was performed using nuclear extracts from untreated (Control NE), NaB-treated (NaB-NE), Scr (Scr-NE) or siATF2 (siATF2-NE) treated K562 cells. Antibody-mediated supershift studies were conducted using NaB-NE with anti-ATF2 (NaB+α-ATF2 Ab) or without ATF2 antibody (NaB+control Ab). The ATF2-containing protein-DNA complex is indicated along with non-specific (NS) bands. (**C**) γ-Globin expression in siATF2 treated cells was determined by RT-qPCR analysis. (**D**) RT-qPCR was performed for RNA samples prepared from K562, pCI-neo and pCI-neo-ATF2 stable cells. Copy number of stably integrated ATF2 was determined using an approach previously published from our lab [20]. (**E**) Western blot (WB) results for K562 and ATF2 stable lines analyzed with anti-FLAG-ATF2 and actin antibodies are shown. The relative γ-globin/GAPD ratio in the ATF2 stable cells was calculated after subtracting the γ-globin/GAPD value produced in the empty vector pCI-neo stable line. (**F**) The pCI-neo-ATF2 stable cells were transfected with siATF2 and western blot was performed (see Materials and Methods). Shown are representative gels with anti-FLAG and actin antibody for two independent experiments. (**G**) RT-qPCR analysis was performed to determine the levels of γ-globin expression after siRNA transfection of the pCI-neo-ATF2 stable line.

To gain insight into the role of ATF2 in γ-globin gene regulation, we first determined the effects of ATF2 knockdown in K562 cells. After siATF2 treatment, RT-qPCR and western blot analysis demonstrated a 50% reduction of ATF2 at the mRNA and protein levels respectively (Fig. 1A). EMSA with a G-CRE probe was performed with siATF2 treated cells. A specific G-CRE-protein complex was observed with K562 nuclear extracts and binding increased after NaB treatment which was supershifted with an ATF2 antibody (Fig. 1B). Furthermore, ATF2 silencing reduced the intensity of the DNA-protein complex indicating a reduction of ATF2 DNA-binding activity. To further corroborate a functional role of ATF2 on γ-globin expression we observed 50–60% decreased expression after siATF2 treatment (Fig. 1C).

To confirm that the observed effect of siATF2 was specific, we created a K562 cell stable line expressing FLAG-tagged ATF2. Fig. 1D shows that ATF2 levels increased two-fold in stable lines compared to empty vector pCI-neo lines after correcting for copy number. Western blot demonstrated enhanced expression of ATF2 protein and a 7-fold increase in γ-globin expression (Fig. 1E). Subsequent siATF2 treatment reduced ATF2 levels (Fig. 1F) and γ-globin expression was repressed 60% (Fig. 1G) indicating that ATF2 positively regulates γ-globin in K562 cells.

### ATF2 Regulates γ-globin Expression in Primary Erythroid Progenitors

**Figure 2:**
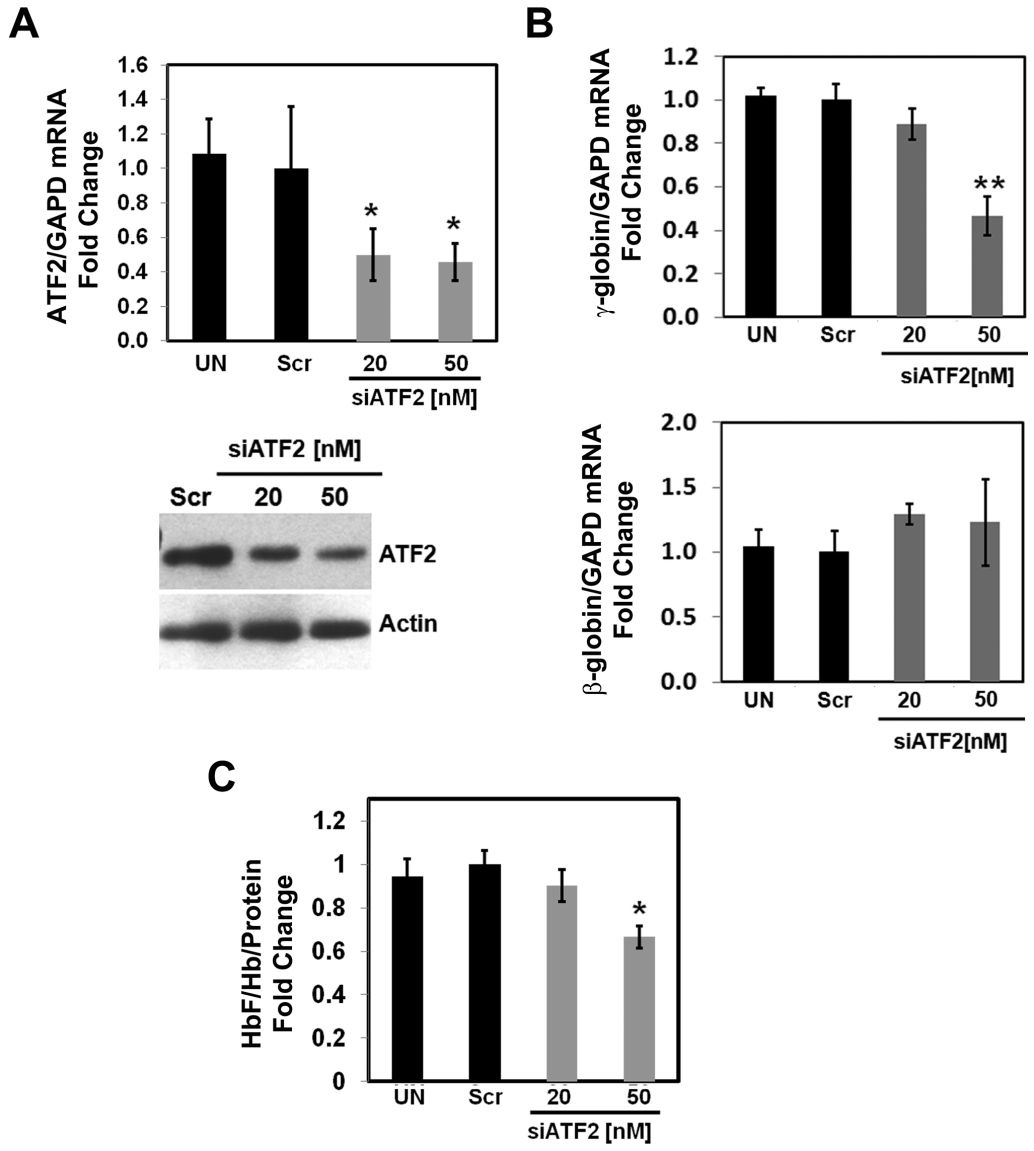
ATF2 is a positive regulator of γ-globin expression in primary cells. Primary erythroid progenitors were cultured in a two-phase liquid system and transfected with Scr siRNA or siATF2. pMaxGFP was used to monitor transfection efficiency on day 11 (Materials and Methods). (**A**) The graph shows RT-qPCR analysis of ATF2 mRNA for three independent experiments. Western blot was conducted with ATF2 and β-actin antibody; a representative gel is shown. Abbreviations: UN, untransfected cells. (**B**) RT-qPCR analysis was completed for γ-globin and β-globin genes expression in primary cells. The fold change of γ-globin/GAPD and β-globin/GAPD was calculated for three independent experiments. (**C**) Cellular lysates from the siRNA-transfected cells were used for ELISA to measure HbF levels. The relative level of HbF/total Hb/total protein was calculated.

We next substantiated the role of ATF2 in γ-globin regulation in human primary erythroid progenitors generated from peripheral blood mononuclear cells as previously published by our group [16]. In this system, the γ-globin to β-globin switch occurs around day 21 [16], serving as an excellent model to determine the effects of ATF2 on γ-globin expression during erythropoiesis. At day 11, when high γ-globin expression occurs ATF2 knockdown produced a 50–60% reduction of ATF2 mRNA and protein in erythroid progenitors (Fig. 2A). Furthermore, siATF2 treatment decreased the γ-globin/GAPD ratio by 55% (Fig. 2B, top graph), whereas the β-globin/GAPD mRNA ratio was not significantly altered (Fig. 2B, bottom graph). We then performed ELISA to measure HbF levels which demonstrated a 30% decrease in HbF level after ATF2 knockdown (Fig. 2C).

Our results demonstrate that ATF2 is a positive regulator of γ-globin in K562 cells and primary erythroid progenitors. Hence, to understand molecular mechanisms of ATF2-mediated γ-globin regulation and the recruitment of the core factors to the G-CRE, we biochemically purify the protein complex exhibiting an ATF2-dependent G-CRE binding activity.

### Purification of G-CRE/ATF2 DNA-binding Partners

**Figure 3:**
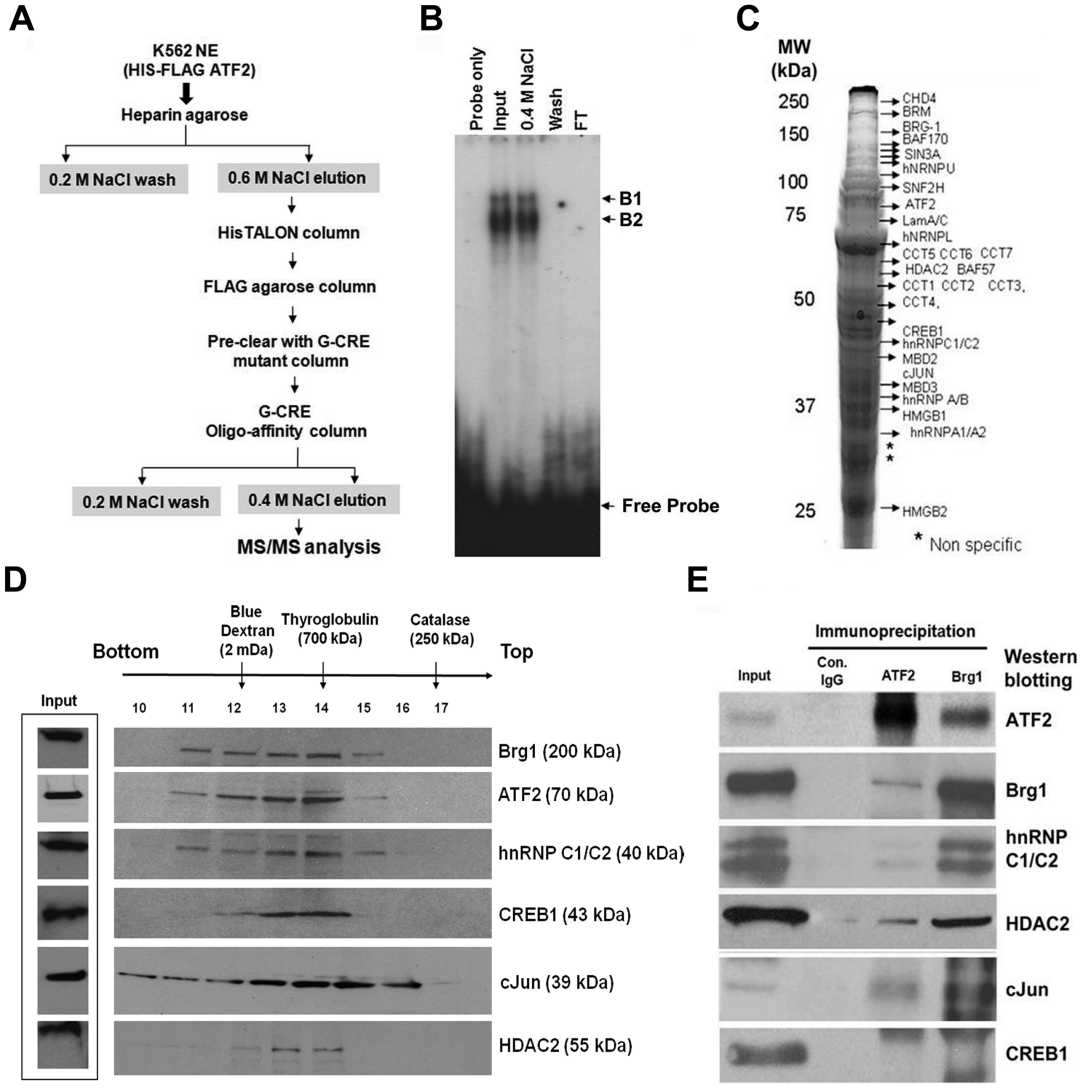
Characterization of the G-CRE multi-protein complex. (**A**) A schematic diagram showing the purification protocol used to identify the G-CRE protein complex using nuclear extracts prepared from pCI-neo-ATF2 stable cells is shown (Material and Methods). Fractions from the 0.6 M NaCl elution were pooled and applied to a HisTALON and subsequently wild-type G-CRE oligonucleotide columns. The 0.4 M NaCl elution from the wild-type G-CRE column was subjected to MS/MS analysis. (**B**) Shown are the results for the EMSA with various protein fractions prior to and after affinity column purification. The following samples were tested: pooled fraction prior to the G-CRE affinity column (Input), pooled fractions at 0.4 M NaCl (0.4 M NaCl), fraction from the 0.2 M NaCl (Wash) and the unbound protein flow through (FT). The arrows indicate the ATF2 complex and non-specific band (NS). (**C**) Proteins associated to the G-CRE were purified, run on a 10% SDS-PAGE gel (shown in image) and stained with colloidal blue; the bands were excised, subjected to trypsin digestion and proteins identified by mass spectrometry. (**D**) Sucrose gradient was performed to demonstrate the association of the G-CRE proteins as a homogenous complex. Ovalbumin (3.5 S, 45 kDa), BSA (4.3 S, 69 kDa), Aldolase (7.3 S, 150 kDa), Catalase (11.3 S, 250 kDa), Thyroglobulin (19.2 S, 700 kDa) and Blue Dextran (2 mDa) were used as standard size markers and loaded in identical parallel gradient. Thirty micro-liters of fractions 10 through 17 were subjected to a 4–15% pre-casted acrylamide gel and analyzed by western blot. (**E**) Nuclear extracts prepared from K562 cells were IP with IgG (control), ATF2 and Brg1 antibodies followed by western blot.

Nuclear extracts prepared from ATF2-K562 stable cell lines were purified through a series of chromatographic columns as shown in Fig. 3A (see Methods and Materials). EMSA results demonstrated two major protein-DNA complexes (B1 and B2) with crude nuclear lysate (input) for the affinity purified product retained in the 0.4 M NaCl elution (Fig. 3B).

Protein fractions eluted off the G-CRE affinity column were loaded on 10% SDS-PAGE gels. A representative image for a colloidal blue-stained gel and proteins identified by MS/MS analysis from the gel slices are shown in Fig. 3C. The proteins identified in the G-CRE complex for untreated and NaB treated extract are summarized in Tables 1 and 2 respectively. We observed the presence of ATF2 and CREB1 along with the chromatin repressive factors such as HDAC1/2, Sin3A, MBD2 and Mi2β in untreated K562 cells. However, after 2 mM NaB treatment, the repressive complex decreased and HMG protein binding become more prominent. We also detected components of T-complex proteins both before and after NaB treatment. The role of the T-complex in γ-globin regulation is of interest however it is beyond the scope of this study.

**Table 1:** Mass spectrometry analysis of protein interacting with G-CRE affinity column from untreated K562 cells.

Trans-acting factors	CREB1 (76), ATF2 (65)
ATP-dependent chromatin remolding complex proteins	BRG1 (34), BAF170 (46), BAF155 (26), BRM (35), BAF57 (66), BAF270 (46), SNF2H (43)
RNA metabolism	hnRNP M (54), hnRNP A1/A3/B (55), hnRNP C1/C2 (65), hnRNP R2 (60), hnRNP U (62), hnRNP H1 (60)
Transcription modulator	HDAC2 (55), HDAC1 (RPD3, 38), Sin3A (52), MBD2 (48), CHD-4 (Mi-2β,44)
RNA polymerase	RNA polymerase 1A (52), RNA polymerase III (55)
Miscellaneous	DNA helicase II, eIF3, LPPRP, TCP1 complex, CD2AP

Functional biological categories of the GCRE protein complex identified by MS/MS analysis. Classifications were made according to the information available in the literature describing the role of these proteins with various DNA/RNA associated functions. The purification procedure is described in Materials and Methods. Numbers in parenthesis indicate the number of peptides detected by MS/MS. Proteins with less than 10 peptide matches were attributed to background contamination and were excluded.

**Table 2:** Mass spectrometry analysis of protein interacting with G-CRE affinity column after NaB treated in K562 cells.

Trans-acting factors	ATF2 (58), CREB1 (65), c Jun (50)
Nuclear Matrix Components	Lamin-A (44), Lamin-B (52), Lamin-C (53)
ATP-dependent chromatin remolding complex proteins	BRG1 (47), BAF170 (56), BAF57 (58)
High mobility group proteins	HMGB1, HMGB2, HMGB3, HMGA1
T-complex protein subunits	CCT1 (55), CCT2 (63), CCT3 (66), CCT4 (54), CCT5 (50), CCT6A (55), CCT7 (60), CCT8 (57)
RNA metabolism	hnRNP A/B, hnRNP C1/C2, hnRNP D, hnRNP G, hnRNP H, hnRNP K, hnRNP L
Miscellaneous	CRKL9 (8), DNMT1 (4), CHD-4 (6), SSRP1 (4)

Functional classification of the GCRE multi-protein complex identified by MS/MS analysis.

To further define the components in the high molecular weight complex, we performed sucrose gradient centrifugation of the proteins eluted from the column and western blot analysis was completed (Fig. 3D). We observed co-migration of Brg1, ATF2, hnRNP C1/C2, CREB1, cJun, and HDAC2 in the fractions corresponding to the molecular mass from 2 mDa and peaking at 700 kDa, implying they associate as a high molecular weight protein complex.

The data from MS/MS analysis and sucrose gradient centrifugation suggest that ATF2 is associated with Brg1 and other proteins in a multi-protein complex. Brg1 is a key member of the SWI/SNF chromatin remodeling complex. To further define which proteins in the G-CRE complex interact with ATF2 and Brg1, co-immunoprecipitation (co-IP) studies were performed with K562 nuclear extracts. Western blot showed that ATF2 co-precipitated with Brg1, hnRNP C1/C2, HDAC2 and cJun (Fig. 3E). Similarly, Brg1 was co-precipitated with ATF2, hnRNP C1/C2, HDAC2, and cJun. Note the abundance of Brg1 and hnRNP C1/C2 in nuclear extracts and the strong interaction between these factors. By contrast, CREB1 was highly scored on the MS/MS analysis (Table 1) but was not detected in association with ATF2 or Brg1 (Fig. 3E). A lack of protein-protein interaction between ATF2/CREB1 has been previously observed [11]. The presence of these proteins at the G-CRE suggests this region undergoes active chromatin remodeling during γ-globin gene activation. These data demonstrated that ATF2, Brg1, hnRNP C1/C2, HDAC2 and cJun interact in a multi-protein complex in K562 cells.

### ATF2 Protein Complex Binds the G-CRE and HS2 of β-locus LCR *in vivo*

**Figure 4:**
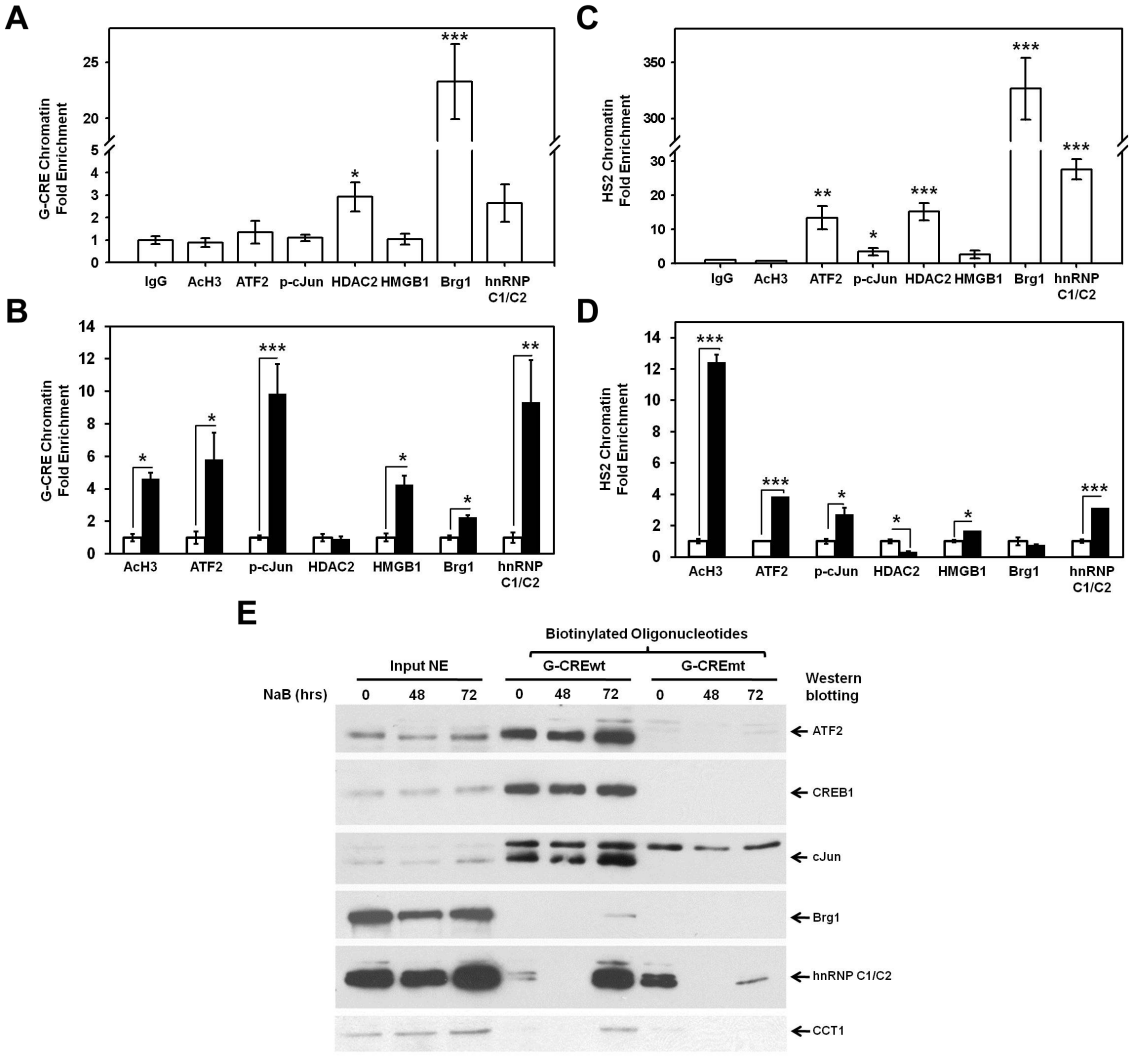
ChIP assay demonstrate *in vivo* binding of protein in the G-CRE complex. K562 cells were treated with 2-chromatin complexes were IP with the antibodies indicated and qPCR analysis completed. (**A**) Shown in the graph is steady-state binding of the factors to the G-CRE in K562 cells. (**B**) The G-CRE chromatin enrichment for NaB-treated samples was studied. The white bars represent untreated and black bars showing data for NaB-treated K562 cells. (**C**) Similar studies were conducted for HS2. Baseline HS2 chromatin fold enrichment is shown in the graph. (**D**) HS2 chromatin enrichment for NaB-treated (black bars) compared to untreated (white bars) cells is shown. (**E**) Oligonucleotides pull-down assay. K562 cells were treated with 2 mM NaB and nuclear extracts prepared. Biotinylated probes were used to pull-down the G-CRE interacting proteins using streptavidin agarose beads. Two oligonucleotides were investigated - wild type G-CRE (G-CREwt) and mutant G-CRE (G-CREmt). Nuclear extracts (50 µg) from the different conditions were loaded as Input NE.

Co-immunoprecipitation results suggest a multi-protein ATF2-containing complex binds the G-CRE *in vitro*, therefore, ChIP studies were performed to explore *in vivo* binding. NaB is a known γ-globin inducer mediated by p38 MAPK signaling previously reported by our laboratory [11], [27]. Thus, we assessed protein interactions of the G-CRE under constitutive and induced conditions. At steady-state, no significant enrichment of ATF2, binding was observed compared to IgG control (Fig. 4A). Acetylated histone H3 at Lys 9 (ac-H3) is an indicator of active transcription hence its enrichment was observed after NaB-induced γ-globin activation. HMGB1 is a high mobility group proteins identified under NaB-treated condition; its binding to DNA causes bending and altered chromatin conformation [28]. At steady-state, neither ac-H3 nor HMGB1 were significantly enriched. By contrast, significant enrichment was produced with Brg1 (22-fold), HDAC2 (2.9-fold) and hnRNP C1/C2 (2.6-fold) antibodies indicating *in vivo* binding of these proteins to the G-CRE. We then determined whether NaB treatment would affect proteins binding to the G-CRE. As shown in Fig. 4B, NaB mediated an open chromatin conformation with enhanced ac-H3 levels and further increased ATF2 and p-cJun binding. Moreover, increased Brg1 and hnRNPC1/C2 binding demonstrates the ability of NaB to modulate several factors bound in the G-CRE multi-protein complex *in vivo*.

The locus control region (LCR) is critical in regulating the expression of the β-like globin genes during erythropoiesis [29]. In particular, HS2 plays an important role in regulating ε- and γ-globin expression during development [30]; therefore we performed studies to determine whether G-CRE target proteins bind HS2. In untreated K562 cells there was chromatin enrichment for Brg1 (325-fold), hnRNP C1/C2 (27-fold), ATF2 (13-fold), HDAC2 (15-fold) and cJun (5-fold) indicating *in vivo* interaction of these proteins with HS2. After NaB treatment marked chromatin opening was supported by enhanced ac-H3 levels and increased ATF2 and p-cJun binding to HS2 (Fig. 4D). We also observed increased HMGB1 and hnRNP C1/C2 binding whereas Brg1 binding was not altered presumably due to high Brg1 binding to HS2 in untreated cells (Fig. 4C). Interestingly, HDAC2-associated HS2 enrichment decreased 70% suggesting that NaB treatment mediates disassociation of a HDAC2-repressor complex.

### Proteins Co-localization in the G-CRE Region

To confirm that the proteins bound to the G-CRE co-localize as a homogenous complex, we performed oligonucleotide pull-down assay with biotinylated wild type (G-CREwt) and mutant (G-CREmt) oligonucleotides (Materials and Methods). We observed simultaneous ATF2, and cJun binding to the G-CREwt probe with untreated K562 extract (Fig. 4E) which was abolished in the G-CREmt probe. Moreover, binding of ATF2 and cJun was increased after NaB treatment. By contrast, Brg1 binding was only detected with the G-CREwt probe after NaB treatment, whereas hnRNP C1/C2 was bound to both probes suggesting possible interaction with nearby sequences and/or other proteins. Interestingly, NaB treatment increased hnRNP C1/C2 to G-CREwt but reduced binding to G-CREmt. These results indicate that ATF2, cJun, Brg1, and hnRNP C1/C2 co-localize to the G-CRE *in vitro* and that mutations in the consensus G-CRE site changed these interactions. Since protein-protein interaction between ATF2 and CREB1 was not detected; these findings suggest different combinations of protein may bind over time and that NaB treatment altered interactions in the G-CRE supporting a role in γ-globin activation by this known HbF inducer.

### Interaction of bZIP Transcription Factors with the G-CRE Correlates with γ- to β-globin Switching Observed in CD34^+^ Cells

**Figure 5:**
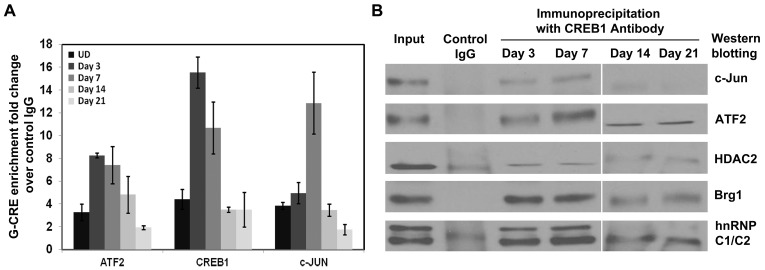
Enrichment of bZIP proteins in the G-CRE during erythroid differentiation. Primary erythroid progenitors were generated in a two-phase *in vitro* expansion culture (Material and Methods). (**A**) ChIP assay was performed using control IgG, ATF2, CREB1 and cJun antibody. Purified chromatin was quantified by qPCR with G-CRE specific primer. ΔΔCT method was used to calculate the fold change of ATF2-, CREB1- and cJun-specific G-CRE enrichment. Results are represented as mean of two biological replicates with each having 2 technical replicates; shown is the mean ± SD. (**B**) Cellular lysates were prepared from primary cells and 50 µg of protein was IP with anti-CREB1 antibody. Control IgG was used as a background control. Precipitants were detected by western blot with the antibodies shown.

To substantiate our *in vitro* findings, we undertook validation of the G-CRE protein complex in human erythroid progenitors. As shown in Fig. 5A, ChIP assay using ATF2, CREB1 and c-Jun antibody demonstrated chromatin enrichment at day 3 and day 5 of erythropoietin-induced erythroid differentiation from CD34^+^ cells. These results confirm our findings that a protein complex regulates γ-globin expression and are recruited to the G-CRE during differentiation. Previous publication shows that γ-globin expression starts at day 3 and peaks at day 5 in this system which corroborates our ChIP results [17]. The recruitment of ATF2, CREB1 and c-Jun to the G-CRE region was lost by day 14 and diminished further by day 21 after γ- to β-globin switching.

To determine interaction between the G-CRE-binding proteins in erythroid progenitors, protein was extracted on day 3, 7, 14 and 21 after EPO-induced differentiation and then co-IP with CREB1 antibody following which western blot was performed. The results (Fig. 5B) showed a strong association of CREB1, c-Jun, HDAC2, Brg1 and hnRNPC1/C2 during the course of erythroid differentiation thereby validating our previous findings that this complex also existed in primary erythroid cells. Of note, in contrast to the sucrose gradient, in co-IP studies we observed interaction between CREB1 and ATF2 in primary erythroid progenitors suggesting CREB1 might participate in the G-CRE complex under normal physiologic conditions. Besides alteration in protein-protein interaction, some of these proteins also showed variation in protein levels by western blot (Figure S1). This data supports highly dynamic binding in this region as erythroid progenitors progressing from fetal to adult hemoglobin production.

### ChIP-seq Confirmation of Proteins Bound to the G-CRE Region

While we were investigating proteins bound to the G-CRE region, the human Encyclopedia of DNA Elements (hENCODE) project published high quality genome-wide ChIP-Seq data [25], [31]. To confirm our results we analyzed the ENCODE data in the Gγ-globin gene region in K562 cells using the UCSC Genome Browser. The single nucleotide polymorphism rs2855122 located within the G-CRE (TGAC**G/A**TCA) was used to indicate the position of G-CRE (Figure S2). We observed transcriptional activity by RNA-seq at the G-CRE and modest levels of H3K4me1 enhancer activity however promoter activity and gene expression related H3K4me3 and H3K9ac marks were not detected. Of note, a switch from H3K4me1 to H3K4me3 was observed near the G-CRE. Along with the active chromatin marks, the G-CRE protein complex members including CREB1, cJun, Brg1, and HDAC2 were bound by ChIP-seq analysis. These results confirm our findings that several bZIP family transcription factor members participate in Gγ-globin regulation. We also investigated the CpG methylation status in brain tissue which demonstrated methylated cytosine at the G-CRE *in vivo* suggesting methylation modulate the interaction between the G-CRE and DNA-binding proteins. Understanding the conditions under which these proteins interact with each other and the G-CRE will determine mechanisms involved in γ-globin expression.

Thus these data suggest that ATF2 and other bZIP family members play an important role in activating γ-globin gene expression and could also be involved in modulating globin gene switching events. Therefore, the identification of ATF2 target genes would be an important step in understanding its role in cellular processes and normal erythropoiesis.

### Identification of ATF2 Downstream Target by Microarray Analysis

**Figure 6:**
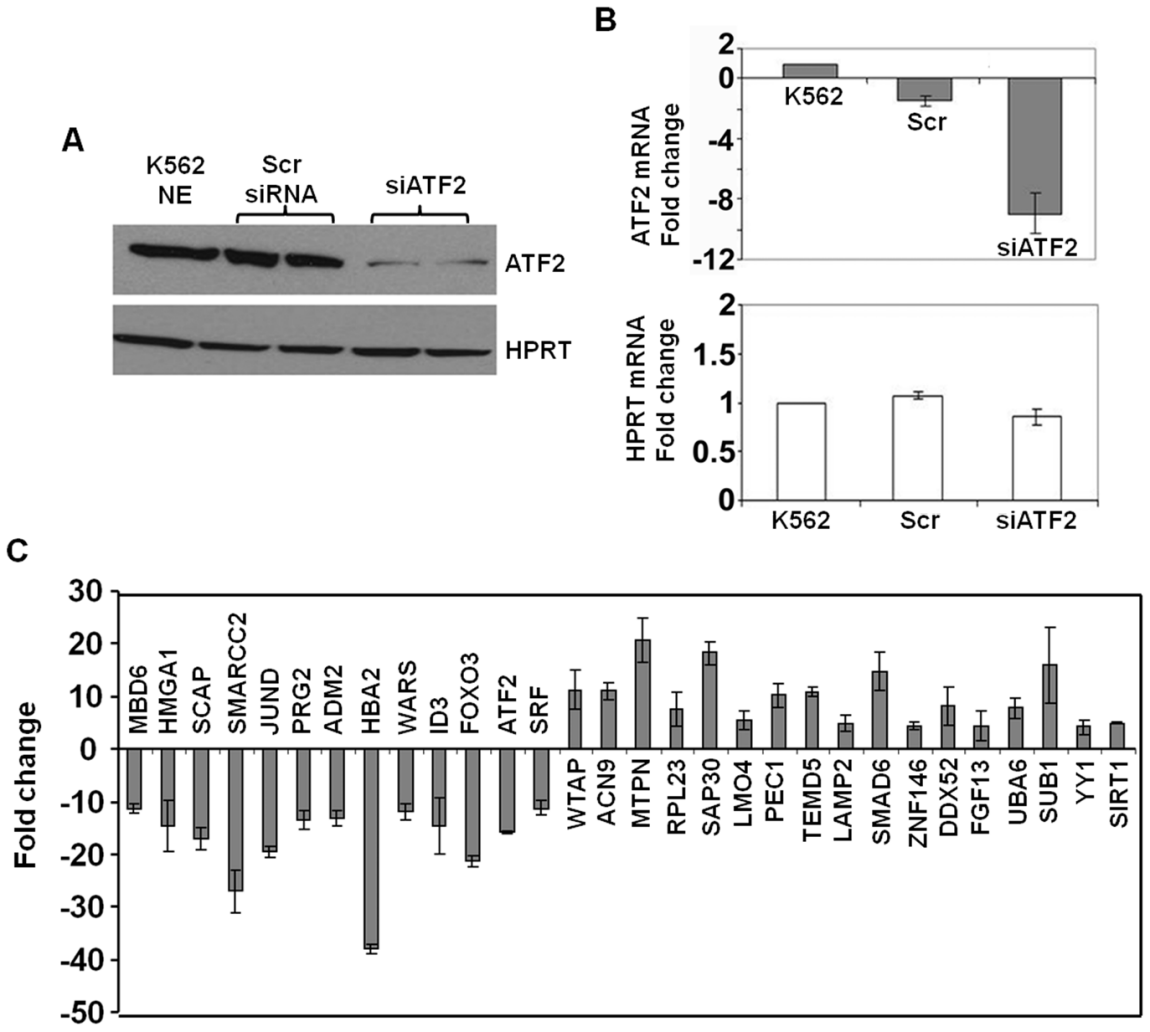
ATF2 gene silencing affects the expression of diverse genes. Microarray analysis was performed on total RNA prepared from Scr siRNA and siATF2 transfected K562 cells. (**A**) Western blot analysis confirmed ATF2 silencing in K562 cells. (**B**) RT-qPCR analysis demonstrated siATF2-mediated target gene silencing without significant change in expression of the housekeeping gene hypoxanthine phosphoribosyltransferase (HPRT1). (**C**) RT-qPCR verification of genes shown to be altered after siATF2 treatment by microarray analysis; gene specific primers are listed in the Table S1. Genes down-regulated by siATF2 treatment (left panel) and genes up-regulated by siATF2 treatment (right panel) are shown.

siATF2 treated K562 cells were used for microarray analysis to identify downstream gene targets in erythroid cells (Fig. 6A and B). Genes with ≥1.2-fold change in expression (p<0.05) between siATF2 and Scr treated cells are shown in Table 3. Subsequent analysis by IPA to evaluate pathway enrichments was completed. The genes down-regulated by siATF2 were enriched for cell death and apoptosis, cellular growth and proliferation, gene expression, and protein synthesis biological pathways (Table 4). Interestingly, we identified 133 genes associated with hematological diseases. The analysis summarized in Table S2 include 109 genes involved in the development of the hematological system and 107 genes with hematopoiesis; differentiation of erythroid cells was among the top functional class affected by siATF2 gene silencing. Next, we examined the 1667 genes up-regulated 0.4–0.8 fold (p<0.05) following siATF2 treatment. Amongst these, 1485 genes were associated with defined biological functions including 31 connected to hematological diseases.

**Table 3:** Genes affected by siATF2 treatment in K562 cells.

Down-regulated Genes	
Fold Change	Numbers of Genes
1.2–1.3	1005
1.3–1.5	899
1.5–1.79	262
1.8–2.3	36
**Up-regulated Genes**	
**Fold Change**	**Numbers of Genes**
0.4–0.6	122
0.6–0.75	813
0.75–0.8	736

**Table 4:** IPA analysis of ATF2-related molecular and cellular functions.

Category	p-value	Number of Genes
Cell Death	5.42E-20–8.09E-03	454
Cellular Growth and Proliferation	2.17E-19–8.39E-03	445
Gene Expression	3.29E-13–8.25E-03	346
Protein Synthesis	1.64E-09–7.32E-03	155
DNA Replication, Recombination, and Repair	2.55E-08–8.36E-03	181

We then confirmed expression patterns by RT-qPCR for a subset of genes identified by microarray analysis. Several down-regulated genes were verified (Fig. 6C) suggesting ATF2 as a positive regulator of SMARCC2, JUND, HBA2, and FOXO3 among others. Similar analysis verified several up-regulated genes indicating a repressive role of ATF2 in modulating MTPN, LMO4, SMAD6, and SUB. Among them, some ATF2 target genes encode transcription factors known to be involved in erythroid or cellular differentiation, such as FOXO3, LMO4, SMAD6, and SMARCC2. We also confirmed down-regulation of the α-like globin genes with ATF2 knockdown suggesting that ATF2 and its family members play a regulatory role in α-globin gene expression as well. Therefore the microarray data and functional analysis suggests that ATF2 might act as a transcription hub in regulating different aspects of hematopoiesis and erythroid maturation.

## Discussion

Transcription of the β-like globin genes during erythroid differentiation is a developmental stage-dependent and tissue-specific process. The LCR located 20 kb upstream of the ε-globin gene plays an important regulatory role by contacting the individual globin promoters through DNA loop formation [32]–[34]. Besides the proximal promoter regions, other *cis*-acting sequences distal to the β-like globin genes have regulatory activity. Studying the roles of these regions in globin gene regulation facilitates in-depth understanding of molecular mechanisms controlling the time- and tissue-specific expression of the β-like globin genes, and has clinical implications for γ-globin reactivation to ameliorate symptoms in β-globin hemoglobinopathy patients.

The G-CRE is a *cis*-acting sequence, located at nucleotide −1222 relative to the transcription start site of Gγ-globin. Three single nucleotide polymorphisms upstream of the Gγ-globin cap site namely rs10126653 at −1450, rs2855121 at −1280 and rs2855122 at −1225 (in the G-CRE) may have functional relevance. Variant genotypes in these three locations result in four pre-Gγ-globin haplotypes associated with different HbF levels in sickle cell anemia patients [35]. The presence of the “G” allele in rs2855122 produced higher transcription rates. Recent publications from our group further demonstrated that the G-CRE interact with CREB1, ATF2 and cJun in response to treatment with NaB [11], [12], [19], [27]. These results strongly suggest the G-CRE plays a role in regulating γ-globin gene expression.

An early report showed that the region between 3′ of ε-globin and 3′ of the ψβ-globin gene is specifically correlated with γ-globin gene expression in the early fetal stage [36]. The above three single nucleotide polymorphisms are located downstream of ε-globin consistent with a potential role of the G-CRE in the formation of a fetal-stage domain. These results were further supported by the ChIP-seq data from the ENCODE project (Figure S2); a high-level of the enhancer H3K4me1 mark at the G-CRE in K562 cells [37]. Also, a switch occurred around the G-CRE from a high H3K4me1 to high H3K4me3 mark suggesting a transition from enhancer to promoter activity. Furthermore, the CpG methylation data in brain tissue demonstrated methylation of target CpG suggesting the interaction of proteins with this region may be altered by changing their methylation status. Therefore, we postulate that the G-CRE and its neighboring sequences may play a role in the creation of a micro-environment required for γ-globin gene expression to facilitate interactions between the γ-globin promoter and LCR. This interpretation requires additional experimental data to be confirmed.

Because the G-CRE is a canonical CRE that interacts with ATF2, experiments were performed to identify its protein binding partners. We identified CREB1 and cJun along with the novel proteins, Brg1, hnRNP C1/C2, HDAC2 and other members of the SWI/SNF chromatin remodeling complex. To demonstrate these proteins were associated as one homogeneous complex we used co-elution through sucrose gradient centrifugation. Subsequent ChIP assay demonstrated Brg1, HDAC2, and hnRNP C1/C2 were highly enriched at the G-CRE and HS2 *in vivo,* confirming the mass spectrometry data.

The role of chromatin remodeling complexes in erythroid development has been demonstrated previously [38]. Moreover, Brg1 interacts with erythroid-specific transcription factors important for regulating chromatin structure, and interaction between the LCR and the downstream β-like globin genes [39]–[45]. Thus, the presence of Brg1 and other members of the SWI/SNF complex in the G-CRE suggest this region may act in concert with the LCR to modulate γ-globin expression. To gain additional evidence for G-CRE function under normal physiological conditions, we performed ChIP assay in primary erythroid cells demonstrating CREB1, ATF2 and c-Jun bind the G-CRE *in vivo*. We presume the G-CRE complex is dynamic in its composition based on the data collected during erythroid differentiation. Subsequently, co-IP studies showed interactions between CREB1 and Brg1, c-Jun, HDAC2 and hnRNP C1/C2. However in contrast to the sucrose gradient results we observed CREB1/ATF2 interactions in primary cells. Although CREB1 does not interact with ATF2 or cJun in K562 cells, its presence in the complex is likely mediated through direct interaction with the G-CRE suggesting different combinations of protein bind the G-CRE over time. We previously demonstrated that CREB1 binds the G-CRE by pull-down assay and EMSA [11] supporting the MS/MS data where CREB1 was bound to the G-CRE. However, we did not observe interaction of CREB1 with Brg1 by co-IP suggesting the presence of the G-CRE site is important for recruiting these proteins together. Despite this difference, given that the γ-globin loci are transcriptionally active in K562 and primary erythroid cells our work clearly demonstrates the role of the G-CRE complex in regulating γ-globin gene expression.

Besides the SWI/SNF complex, we also identified many heterogeneous nuclear ribonucleoproteins. In general, these are RNA binding proteins, however, hnRNP C1/C2 bind a sequence-specific DNA recognition motif in the HS2 core to form a LCR-associated remodeling complex [44]. Moreover, identification of several HMG factors associated with the G-CRE in NaB-treated cells suggests that these proteins may facilitate conformational changes. It remains to be determined if conformational changes occur in primary erythroid cells to enhance γ-globin expression.

When determining the effect of NaB treatment on proteins associated with the G-CRE (Table 1 and 2), we observed a disappearance of HDACs and the methyl-CpG-binding-domain protein. Normally, these proteins are present in diverse silencing complexes to repress gene transcription by binding to methylated CpG sites [46]. ENCODE analysis indicates that the CpG dinucleotide in the G-CRE site is partially methylated in brain tissue suggesting there may exist a transcription repressor complex associating with the G-CRE at steady-state. It is known that NaB treatment leads to ATF2 and cJun activation through p38 MAPK-mediated phosphorylation. We speculate this activation may cause the displacement of the repressor complex from the G-CRE and enhance the binding of ATF2 to the region. This interpretation is supported by decreased *in vivo* association of HDAC2 in the G-CRE and HS2 after NaB treatment.

Various transcription factors, such as GATA1, GATA2, NFE2, EKLF and BCL11A, have been implicated in the switch from γ-globin to β-globin expression during development [47]–[49]. Although ATF2 is a ubiquitous transcription factor, a conditional mutant of ATF2/ATF7 demonstrated increased apoptotic hematopoietic cells [50]. Moreover, gene profiling in primary erythroid progenitors by our group and others [3], [51] support a role of CREB1/ATF2 in regulating erythroid differentiation. These findings are consistent with our results that ATF2 interaction with various bZIP family members and other G-CRE complex proteins erythroid progenitors. Moreover, the ChIP assay results for ATF2, CREB1 and cJun in erythroid progenitors provide further evidence for a role of these proteins in globin gene regulation during erythroid differentiation. Previous transcriptome analysis from the Pace laboratory [3] demonstrated that ATF2 levels are high throughout erythropoiesis derived from adult CD34^+^ stem cells, similar to that reported by Merryweather and colleagues [51]. Similarly ATF2 expression remains high during umbilical cord blood CD34^+^ stem cells derived erythropoiesis (personal communication Betty Pace). These data are consistent with a regulatory role of ATF2 in the various stages of erythropoiesis.Finally a more global role of ATF2 in hematopoiesis is supported by the potential downstream target genes identified by the microarray analysis after ATF2 knockdown in K562 cells. Besides the genes previously reported [52] we identified new targets such as SMARCC2, LMO4, YY1, GATA1, HBA2, and ALAS2. The latter genes, ALAS2 and HBA2, suggest a role of ATF2 in regulating heme biosynthesis and α-globin expression respectively which are also essential for normal hemoglobin production. With the evidence provided in this study, we postulate that ATF2 is an important transcription factor affecting many aspects of the cellular functions related to erythropoiesis. Further investigation on the dynamic nature of the G-CRE multi-protein complex, correlated with chromatin structure in the G-CRE region in primary erythroid cells, will shed light on molecular mechanisms of γ-globin gene expression and hemoglobin switching.

## Supporting Information

Figure S1 **Western blot analysis for the G-CRE-interacting proteins in CD34^+^ cells treated with erythropoietin.** Whole cell lysates were prepared from CD34^+^ with erythropoietin at indicated days. Western blot analyses were performed with antibody specific for cJun, ATF2, HDAC2, Brg1, hnRNP C1/C2 and CREB1. Shown is a representative image of two independent experiments.
(TIF)

Figure S2 **ENCODE data demonstrate co-localization of G-CRE complex proteins in K562 cells.** ChIP-seq data for the tracks indicated at the right side of the figure were generated using the UCSC Genome Browser for the genomic region 5,273,486–5,279,090 (GRCh37/hg19) on chromosome 11. The Gγ-globin gene (HBG2) is indicated with the arrows showing the direction of transcription. Five SNPs in the 5′ region of Gγ-globin are shown with the respective SNP identification numbers; SNP rs2855122 resides within the G-CRE (red bar). The numbers on the right side indicate the maximum z-scores showing the strength of the signals. Negative control track for ChIP-seq is also shown (Inpt). An arrow on the MeDIP track indicates the position of rs2855122. The red box indicates the changes in DNA-binding protein interactions and chromatin marks in the G-CRE.
(TIF)

Table S1 **Sequences of primers used for qPCR.**
(DOC)

Table S2 **Involvement of ATF2-mediated down-regulated genes in hematopoiesis.**
(DOC)
