# Supplementary figures and images for: Regulation of Gγ-Globin Gene by ATF2 and Its Associated Proteins through the cAMP-Response Element
Source: PLoS One. 2013 Nov 6;8(11):e78253. doi: 10.1371/journal.pone.0078253 (PMC3819381; doi:10.1371/journal.pone.0078253)

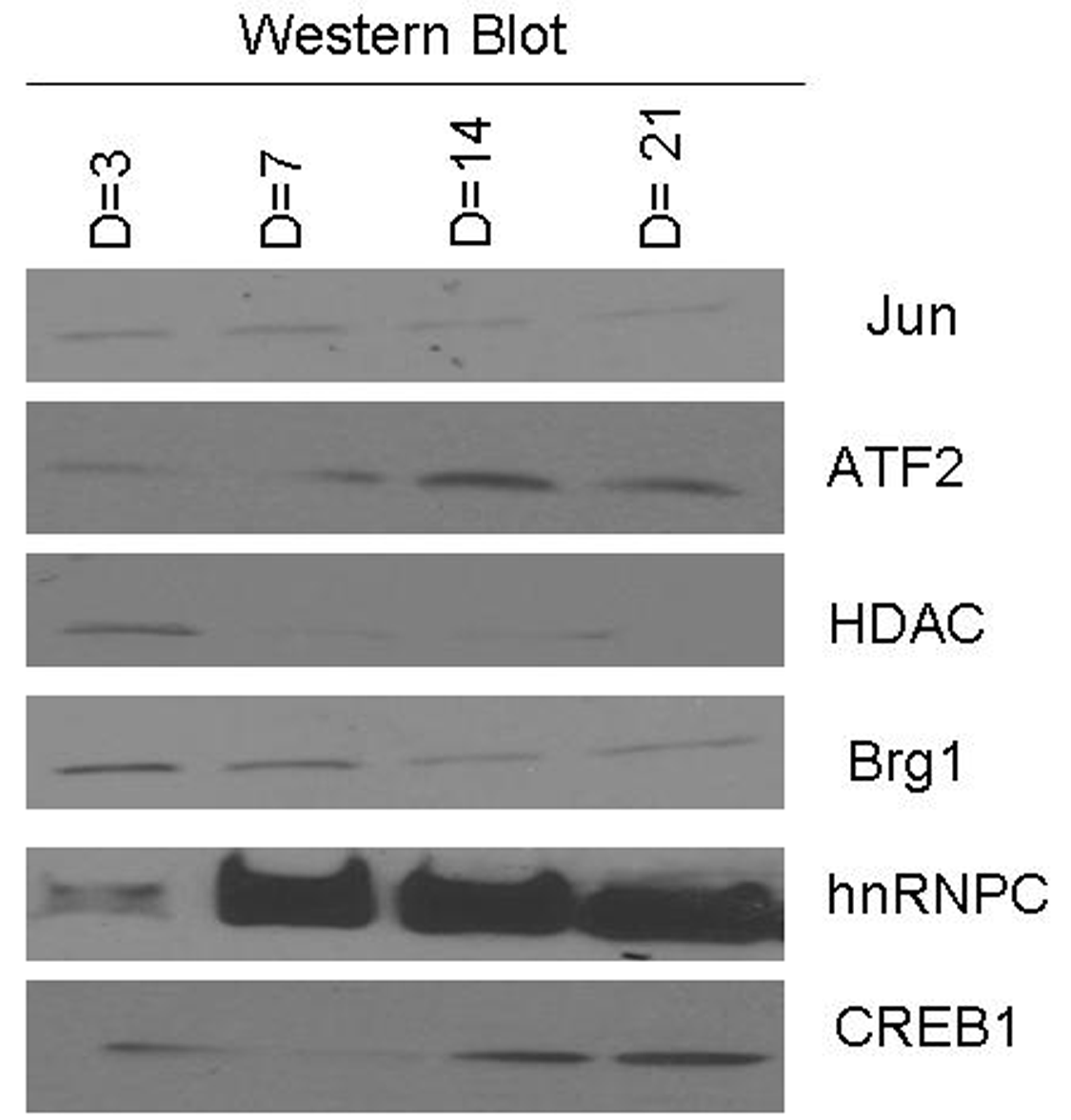

Supplement: Figure S1 — Western blot analysis for the G-CRE-interacting proteins in CD34+ cells treated with erythropoietin. Whole cell lysates were prepared from CD34+ with erythropoietin at indicated days. Western blot analyses were performed with antibody specific for cJun, ATF2, HDAC2, Brg1, hnRNP C1/C2 and CREB1. Shown is a representative image of two independent experiments. (TIF) [file pone.0078253.s001.tif]

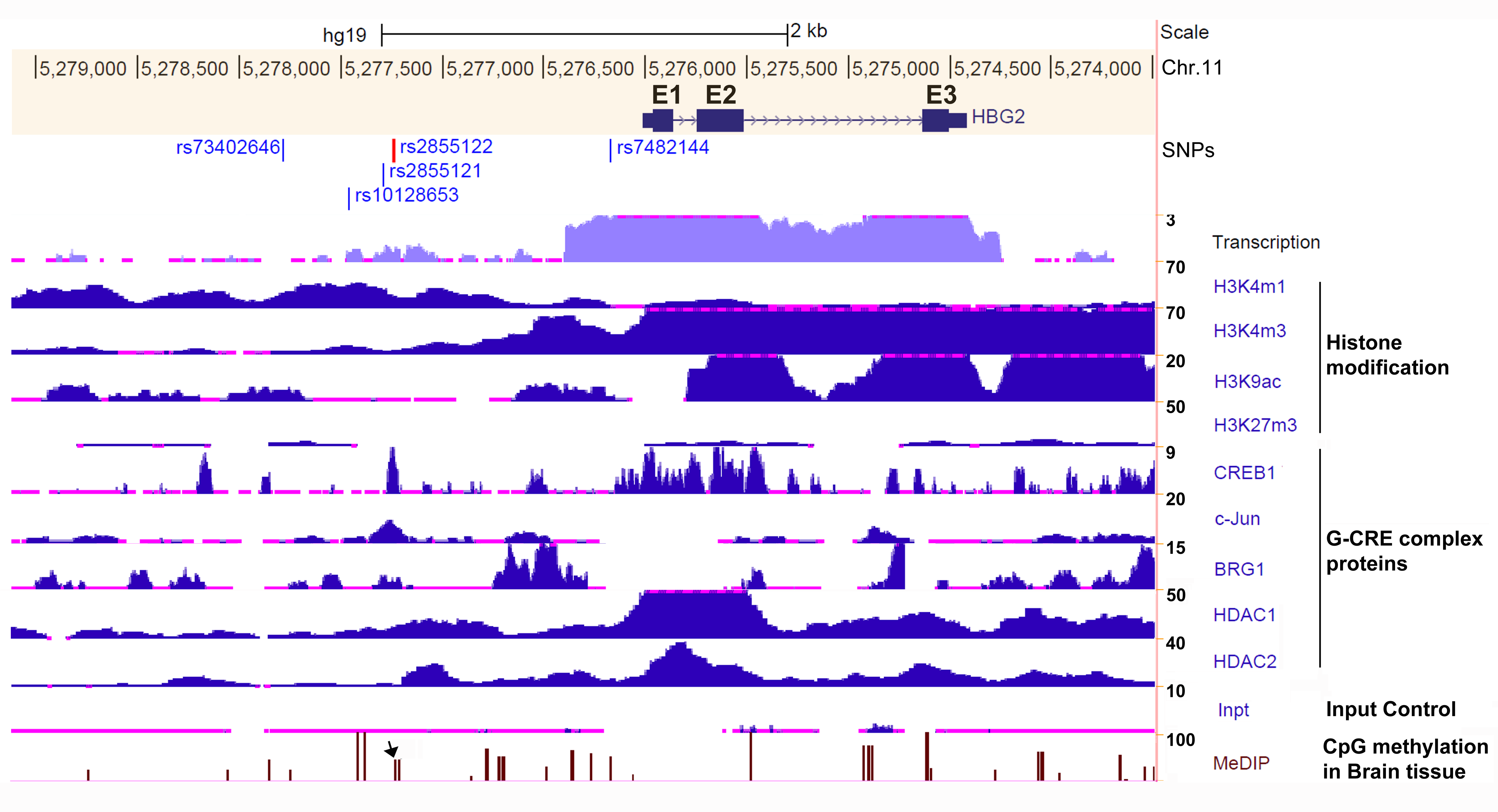

Supplement: Figure S2 — ENCODE data demonstrate co-localization of G-CRE complex proteins in K562 cells. ChIP-seq data for the tracks indicated at the right side of the figure were generated using the UCSC Genome Browser for the genomic region 5,273,486–5,279,090 (GRCh37/hg19) on chromosome 11. The Gγ-globin gene (HBG2) is indicated with the arrows showing the direction of transcription. Five SNPs in the 5′ region of Gγ-globin are shown with the respective SNP identification numbers; SNP rs2855122 resides within the G-CRE (red bar). The numbers on the right side indicate the maximum z-scores showing the strength of the signals. Negative control track for ChIP-seq is also shown (Inpt). An arrow on the MeDIP track indicates the position of rs2855122. The red box indicates the changes in DNA-binding protein interactions and chromatin marks in the G-CRE. (TIF) [file pone.0078253.s002.tif]
