# Supplementary material for: Regulation of Gγ-Globin Gene by ATF2 and Its Associated Proteins through the cAMP-Response Element
Source: PLoS One. 2013 Nov 6;8(11):e78253. doi: 10.1371/journal.pone.0078253 (PMC3819381; doi:10.1371/journal.pone.0078253)
Supplement: Table S1 — Sequences of primers used for qPCR. (DOC) [file pone.0078253.s003.doc]

| **Table S1. Sequences of primers used for qPCR** | | |
| --- | --- | --- |
| **Gene/Loci** | **Forward Primer** | **Reverse Primer** |
| Gene Expression | | |
| γ-globin | 5’ GGCAACCTGTCCTCTGCCTC 3’ | 5’GAAATGGATTGCCAAAACGG 3’ |
| β-globin | 5’ CTCATGGCAAGAAAGTGCTCG 3’ | 5’ AATTCTTTGCCAAAGTGATGG 3’ |
| GAPD | 5’ GAAGGTGAAGGTCGGAGT 3’ | 5’ GAAGATGGTGATGGGATTTC 3’ |
| MBD6 | 5’ ATGGGGGACACGTGGTGGCT 3’ | 5’ TCAGAATGGGGCCCGGGAGG 3’ |
| HMGA1 | 5’ CCTGAGTGACACGGCTGGCG 3’ | 5’ CGCCGCTGGTAGCAAATGCG 3’ |
| SCAP | 5’ ACGCTGTACAAGGTGGCGGC 3’ | 5’ CGGGTGGCGCATAGCCGTAG 3’ |
| SMARCC2 | 5’ AGTATTAACCTCCCCGCTCCTCCT 3’ | 5’ GTGGTCGCCGGCAGGTTAGG 3’ |
| JUND | 5’ CGACAAGCTGGACCCCCTGC 3’ | 5’ GCGCGGTGTACAAAGGGGCA 3’ |
| PRG2 | 5’ GACGGCAGCCGCTGGAACTT 3’ | 5’ ACTGCTGGCTGGGACCAGCT 3’ |
| ADM2 | 5’ AAGCTTCACCGGGCCCTCCA 3’ | 5’ CCCAGCACACAGCCCACTCG 3’ |
| HBA2 | 5’ ATAAACCCTGGCGCGCTCGC 3’ | 5’ GCCTCCGCACCATACTCGCC 3’ |
| WARS | 5’ CCGGCTGGGCAGGGATGAAATT 3’ | 5’ CTGGTAGGTGCTGGGTTCCCTGG 3’ |
| ID3 | 5’ ACTGGCAGGGGGCAGGGAAG 3’ | 5’ GGTCTGACGCCGAAGACCGC 3’ |
| FOXO3 | 5’ CGGCCGCCGACTCCATGATC 3’ | 5’ CCGGGCCGAGTCCTCAAGGA 3’ |
| ATF2 | 5’ ATTGCCCTGTAACCGCCATGCAG 3’ | 5’ GTCCGCCATCTGGGTGAGGAC 3’ |
| SRF | 5’ GCCATTCCCCAACGGGGTGG 3’ | 5’ GCAGCTGCATGCCTGGGACA 3’ |
| NXF1 | 5’ CCTACATCAGCGCCATTCGCGAA 3’ | 5’ TTCCCTTGCAGGGCGGTAACG 3’ |
| ACN9 | 5’ GGCCTGTGTCGTCAGGACGC 3’ | 5’ AAACCCAAGCTCCGCTGCGG 3’ |
| MTPN | 5’ GCGGAACCTCTCTGCTGGGC 3’ | 5’ GATGAGGAGGCGGTGGCAGC 3’ |
| RPL23 | 5’ GGTGGGCGGGGCGTTAAAGT 3’ | 5’ CCGGAATTTCGCACCAGAGGACC 3’ |
| SIN3A | 5’ GCCAGCAGAGACCAGCAGGC 3’ | 5’ CTCGTCAGGCGTGAAGCCGT 3’ |
| LMO4 | 5’ GCCGCCGCCTCTCGCAATAT 3’ | 5’ AGCGGTCCGCAATCTTGCCC 3’ |
| LAMP2 | 5’ ATCTCAGCTACTGGGATGCCCCC 3’ | 5’ CCCACCGCTATGGGCACAAGG 3’ |
| SMAD6 | 5’ GGGCCCGAATCTCCGCCAC 3’ | 5’ TGGCGTCTGAGAATTCACCCGGA 3’ |
| ZNF146 | 5’ GTGGCGCGAAAGTAGGAGGAAGA 3’ | 5’ CTTCCGCGCAGATCCTTCCCAC 3’ |
| DDX52 | 5’ CGCCGTAGAACTGTGGCGCTT 3’ | 5’ TCTGGAATCGAGCTGCGTCTGC 3’ |
| FGF13 | 5’ TCCCAAGTCCGACCCGCTCG 3’ | 5’ TTCCATCCGCCTGCAGCTGC 3’ |
| UBA6 | 5’ TCCTACCTTCCAGTAGCCGGCG 3’ | 5’ TTATTTGTGCTGCCAGTCCCCCA 3’ |
| SUB1 | 5’ CAGTCGCGAGCGAACGACCA 3’ | 5’ AGGGCTCTCGAAGTCTCACCTG 3’ |
| YY1 | 5’ AGGGCGAACGGGCGAGTGG 3’ | 5’ ACTCCTCAACCCGAGCCCAGA 3’ |
| SIRT1 | 5’ TGACTGGACTCCAAGGCCACGG 3’ | 5’TCAGGTGGAGGTATTGTTTCCGGCA 3’ |
| HPRT1 | 5' CGACGAGCCCTCAGGCGAAC 3' | 5' CGGGTCGCCATAACGGAGCC 3' |
| ChIP assay | | |
| G-CRE region | 5'AAGCCTTACACAGGATTATGAAGTCTG 3' Coordinates: 41512 - 41538 | 5' ACATGGCAGGAAGTATTCATGCTG 3' Coordinates: 41739 - 41762 |
| HS2 | 5' CCTTCTGGCTCAAGCACAGC 3' Coordinates: 16849 - 16868 | 5' ATAGGAGTCATCACTCTAGGC 3' Coordinates: 16926 - 16946 |
| Coordinates for the ChIP assay primers are based on the HBB record NG_000007 in GenBank | | |
