# Supplementary material for: Regulation of Gγ-Globin Gene by ATF2 and Its Associated Proteins through the cAMP-Response Element
Source: PLoS One. 2013 Nov 6;8(11):e78253. doi: 10.1371/journal.pone.0078253 (PMC3819381; doi:10.1371/journal.pone.0078253)
Supplement: Table S2 — Involvement of ATF2-mediated down-regulated genes in hematopoiesis. (DOC) [file pone.0078253.s004.doc]

| **Table S2. Involvement of ATF2-mediated down-regulated genes in hematopoiesis** | | | | |
| --- | --- | --- | --- | --- |
| **Category** | **Function** | **Function Annotation** | **p-value** | **Molecules** |
| Hematopoiesis | Maturation | Maturation of Red Blood Cells | 3.40E-06 | ABL1, ALOX15, BCL2L1, CDKN1A, CLEC11A, E2F2, FOXO3, G6PD, GATA1, TAL1, ZFPM1 |
|  |  | Maturation of Erythroid Progenitor Cells | 8.77E-06 | ABL1, ALOX15, BCL2L1, CDKN1A, CLEC11A, E2F2, FOXO3, GATA1, ZFPM1 |
|  |  | Maturation of Erythroid Cells | 3.59E-04 | ABL1, BCL2L1, CLEC11A, FOXO3, GATA1, ZFPM1 |
|  |  | maturation of hematopoietic progenitor cells | 1.76E-03 | ABL1, ALOX15, BCL2L1, CDKN1A, CLEC11A, E2F2, FOXO3, GATA1, PML, RARA, TAL1, ZFPM1 |
|  |  | maturation of erythroblasts | 5.23E-03 | CDKN1A, E2F2, GATA1 |
|  |  | maturation of granulocytes | 6.94E-03 | ACHE, PML, RARA, STAT3 |
|  | differentiation | differentiation of megakaryocytes | 5.25E-06 | BAIAP2, E2F1, GATA1, HIST1H4A (includes others), HMGA1, KDM1A, L3MBTL1, MYB, RPS6KA1 (includes EG:20111), SP1, TAL1, WASF2, ZFPM1 |
|  |  | differentiation of hematopoietic cells | 1.99E-05 | ABL1, BAIAP2, BCL2L1, BMP2, BSG (includes EG:12215), BTG2, CLEC11A, CTBP1, CTNNB1, E2F1, EPAS1, GATA1, HIST1H4A (includes others), HMGA1, IL15 (includes EG:16168), JUNB, KDM1A, L3MBTL1, LONP1, MAD1L1, MAX, MYB, MZF1, NFYA, PICALM, PML, PRKDC, PTPN1, RARA, RPS6KA1 (includes EG:20111), RXRA, SHH, SMARCB1, SP1, SYK, TAL1, TCF3, TYK2, VEGFA, WASF2, ZFPM1 |
|  |  | differentiation of hematopoietic progenitor cells | 3.84E-05 | ABL1, BAIAP2, BCL2L1, BMP2, BSG (includes EG:12215), BTG2, CLEC11A, CTBP1, CTNNB1, E2F1, EPAS1, GATA1, HIST1H4A (includes others), HMGA1, IL15 (includes EG:16168), JUNB, KDM1A, L3MBTL1, LONP1, MAD1L1, MAX, MYB, NFYA, PICALM, PML, PRKDC, PTPN1, RARA, RPS6KA1 (includes EG:20111), RXRA, SHH, SMARCB1, SP1, SYK, TAL1, TCF3, VEGFA, WASF2, ZFPM1 |
|  |  | differentiation of red blood cells | 2.30E-04 | ABL1, ALAS2, BCL2L1, EPAS1, FOXO3, GATA1, ID2, JUNB, KDM1A, MAPK14, MAX, MLL5, MYB, NCKAP1L, PTPN1, RPS14, RXRA, TAL1, TCEA1, VEGFA, ZFPM1 |
|  |  | differentiation of bone marrow cells | 9.26E-04 | BAIAP2, BCL2L1, BMP2, CTBP1, CTNNB1, E2F1, GATA1, HIST1H4A (includes others), HMGA1, IL15 (includes EG:16168), KDM1A, L3MBTL1, MYB, NFE2L1, NFYA, PML, RARA, RPS6KA1 (includes EG:20111), SMARCB1, SP1, TAL1, VEGFA, WASF2, ZFPM1 |
|  |  | differentiation of erythroid cells | 5.09E-03 | ABL1, BCL2L1, GATA1, JUNB, MAX, MYB, PTPN1, RXRA |
|  |  | differentiation of promyelocytes | 7.08E-03 | PML, RARA |
|  | developmental process | developmental process of red blood cells | 2.23E-05 | ABL1, ALAS2, ALOX15, BCL2L1, CDKN1A, CLEC11A, E2F2, E2F4, EIF2C2, EPAS1, FOXO3, G6PD, GATA1, HBA1/HBA2, ID2, IRF1 (includes EG:16362), JUNB, KDM1A, MAPK14, MAX, MLL5, MYB, NCKAP1L, NFYA, PTPN1, RPS14, RXRA, TAL1, TCEA1, VEGFA, ZFPM1 |
|  | erythropoiesis | erythropoiesis | 5.05E-05 | ABL1, ALAS2, ALOX15, BCL2L1, CDKN1A, CLEC11A, E2F2, E2F4, EIF2C2, EPAS1, FOXO3, G6PD, GATA1, HBA1/HBA2, HMGA1, ID2, IRF1 (includes EG:16362), JUNB, KDM1A, MAPK14, MAX, MLL5, MYB, NCKAP1L, NFYA, PTPN1, RPS14, RXRA, TAL1, TCEA1, VEGFA, ZFPM1 |
|  | myelopoiesis | myelopoiesis | 5.11E-05 | ABL1, ACHE, ALAS2, ALOX15, BCL2L1, CDKN1A, CLEC11A, E2F2, E2F4, EIF2C2, EPAS1, FOXO3, G6PD, GATA1, HBA1/HBA2, HIST1H4A (includes others), HMGA1, ID2, IRF1 (includes EG:16362), JUN, JUNB, KDM1A, MAD1L1, MAPK14, MAX, MLL5, MYB, NCKAP1L, NFYA, OGT, PML, PTPN1, RALGDS, RARA, RPS14, RXRA, STAT3, TAL1, TCEA1, VEGFA, ZFPM1 |
|  | hematopoiesis | hematopoiesis | 6.31E-04 | ABL1, ACHE, AKT1, AKT2, ALAS2, ALOX15, BAX, BCL2L1, BMP2, BSG (includes EG:12215), CARD11, CDK2, CDKN1A, CDKN2D, CLEC11A, CNN2, CSK, CTBP1, CTF1, CTNNB1, CTNNBIP1, DOCK2, E2F1, E2F2, E2F4, EEF1A2, EIF2C2, ELF4, ELK1, EPAS1, EZH2, FCGR2A, FKBP1A, FOXO3, G6PD, GAB2, GATA1, GRAP2, GRB2, HBA1/HBA2, HIST1H4A (includes others), HMGA1, HMGB3, ID2, ID3 (includes EG:15903), IGLL1/IGLL5, IL11RA, IL15 (includes EG:16168), IRF1 (includes EG:16362), IRF3, ITGA4, JAG2, JUN, JUNB, KDM1A, LAMP1, LONP1, MAD1L1, MAP2K2, MAPK14, MAPKAP1, MAX, MCL1, MLL5, MYB, MZF1, NCAPH2, NCKAP1L, NFYA, NKX2-3, NKX2-5, OGT, PICALM, PLP1 (includes EG:18823), PML, PRKDC, PRKX, PTPN1, RALGDS, RARA, RNF128, RPA1, RPS14, RXRA, SHH, SMAD2, SRF, STAT3, SYK, TAL1, TAPBP, TAZ, TCEA1, TCF3, TGFBR1, TXNRD2, TYK2, VEGFA, ZFPM1 |
|  | hematopoiesis | hematopoiesis of cells | 6.96E-04 | ACHE, CLEC11A, CNN2, CTNNB1, EPAS1, GATA1, HIST1H4A (includes others), HMGA1, IRF1 (includes EG:16362), MAPK14, MCL1, MZF1, NFYA, NKX2-5, PICALM, PML, RARA, RPA1, TAL1, TAZ, TCF3, TXNRD2, ZFPM1 |
|  | hematopoiesis | hematopoiesis of leukemia cell lines | 7.08E-03 | HIST1H4A (includes others), MAPK14 |
|  | development | development of blood cells | 4.42E-03 | ABL1, ACHE, AKT1, AKT2, BAX, BCL2L1, BSG (includes EG:12215), CARD11, CDK2, CDKN1A, CLEC11A, CNN2, CSK, CTNNB1, DOCK2, E2F1, E2F4, EEF1A2, EIF2C2, ELF4, ELK1, EPAS1, EZH2, FCGR2A, FKBP1A, FOXO3, G6PD, GATA1, GRAP2, GRB2, HBA1/HBA2, HMGA1, ID2, ID3 (includes EG:15903), IGLL1/IGLL5, IL11RA, IL15 (includes EG:16168), IRF1 (includes EG:16362), IRF3, ITGA4, JAG2, JUNB, LAMP1, LONP1, MAD1L1, MAP2K2, MAPK14, MAPKAP1, MYB, MZF1, NCAPH2, NCKAP1L, NFYA, NKX2-5, PICALM, PLP1 (includes EG:18823), PRKDC, RARA, RNF128, RPA1, SHH, SMAD2, SRF, STAT3, SYK, TAL1, TAPBP, TAZ, TCF3, TGFBR1, TXNRD2, TYK2, VEGFA, ZFPM1 |
|  | selection | selection of thymocytes | 7.43E-03 | CARD11, DOCK2, ELK1, GRB2, ID3 (includes EG:15903), JAG2, MAD1L1, SHH |
